# Improving Outcomes: Early Nursing Functional Evaluation of Patients with Intracranial Aneurysms After Surgery—Preliminary Results

**DOI:** 10.3390/medicina61111986

**Published:** 2025-11-05

**Authors:** Dominika Bąk, Katarzyna Kwiecień-Jaguś, Renata Piotrkowska, Monika Kopeć

**Affiliations:** 1 The Surgical Nursing Scientific Club at the Department of Surgical Nursing, Medical University of Gdansk, 80-211 Gdansk, Poland; dombak@gumed.edu.pl; 2 Department of Anaesthesiology Nursing & Intensive Care, Faculty of Health Sciences, Medical University of Gdansk, 80-211 Gdansk, Poland; 3 Department of Surgical Nursing, Faculty of Health Sciences, Medical University of Gdansk, 80-211 Gdansk, Poland; renata.piotrkowska@gumed.edu.pl; 4 Department of Human Nutrition, University Warmia and Mazury, 10-718 Olsztyn, Poland; monika.kopec@uwm.edu.pl

**Keywords:** intracranial aneurysm, functional outcome, postoperative, assessment

## Abstract

Background and Objectives: Intracranial aneurysms are localized, pathological dilatations of the walls of cerebral arteries. While small, unruptured aneurysms may not require intervention, treatment decisions involve complex clinical dilemmas, balancing the risks of treatment with the possibility of neurological deficits. This study aimed to functionally evaluate patients after treatment (embolization or craniotomy) of unruptured intracranial aneurysms. Material and Methods: A total of 75 patients from one of the hospitals in Northern Poland were included in the study. The study was retrospective, with data analysis covering the period from December 2023 to October 2024. Data were collected using a diagnostic survey based on electronic medical records, including sociodemographic and clinical data. Assessments using the Barthel index, Rankin scale, NRS scale, and Waterlow scale were performed on days 1 and 3 postoperatively. Statistical analysis included descriptive statistics (n, %, mean, median, SD, IQR), tests of independence (χ2) and comparisons (Wilcoxon, Mann–Whitney, Kruskal–Wallis, Dunn). Results: A significant part of the patients were women (78.67%). Most were aged 66–75. Embolisation was the primary treatment method (85.33%), resulting in statistically significantly better functional results than clipping on the 1st and 3rd postoperative days. A statistically significant relationship was found between higher Waterlow scale scores and an increased risk of complications on the first and third days after the procedure. Conclusions: The study indicates that embolisation was associated with better early functional outcomes than clipping. Given the non-randomized treatment allocation, this association may reflect both the procedure type and underlying patient or aneurysm characteristics. The Waterlow score successfully predicted the risk of postoperative complications. The results highlight the importance of early and comprehensive postoperative evaluation to optimize patient care. The results reflect early postoperative recovery (days 1 and 3) and should not be directly extrapolated to long-term functional outcomes.

## 1. Introduction

An intracranial aneurysm is a local, pathological dilatation of the cerebral arterial wall that occurs when the vessel’s structure is weakened, leading to its bulging [1]. The risk factors for a brain aneurysm are varied. They include non-modifiable factors such as age, gender, family history, comorbidities, including cystic kidney disease and Marfan syndrome, or modifiable factors such as hypertension and smoking [2].

Small, unruptured intracranial aneurysms often do not require medical intervention beyond conservative treatment and observation with imaging tests such as cerebral angiography. The decision to treat unruptured aneurysms is complex and always creates a clinical dilemma. The risks of treatment and the potential for permanent neurological deficits and even death must be considered [3]. Despite the advancement of treatment methods, patients, after both endovascular surgery and surgical aneurysm clipping, are at risk of developing several complications like neurological limitations after the medical intervention, Waterlow, Glasgow and mRS evaluation result. The early postoperative period is crucial for assessing patients’ functional status and enabling them to return to as much normal fitness as quickly as possible. Recovery during this time should include close monitoring of the patient for neurological symptoms, vital signs, and pain assessment. It is also essential to introduce rehabilitation exercises at an early stage. The topic of the presented work is critical due to the high risk of complications related to the procedure. Early functional assessment enables faster detection of potential problems, the implementation of appropriate interventions, and improved quality of life after surgery. Research by other authors shows that patients who survive treatment often experience cognitive, emotional, and functional limitations. There are very few original research studies led by nursing personnel evaluating patient condition during the three days after the surgery. Most of the work provides information on patients’ clinical characteristics and the need to follow up on their condition, as recurrence rates are estimated at 10–30% [4]. However, patients’ functional recovery depends not only on the clinical condition but also on nursing care during the postoperative period.

This study aims to assess early functional outcomes after treatment with embolization or traditional craniotomy for unruptured intracranial aneurysms.

## 2. Materials and Methods

### 2.1. Study Design and Inclusion and Exclusion Criteria

**Figure 1:**
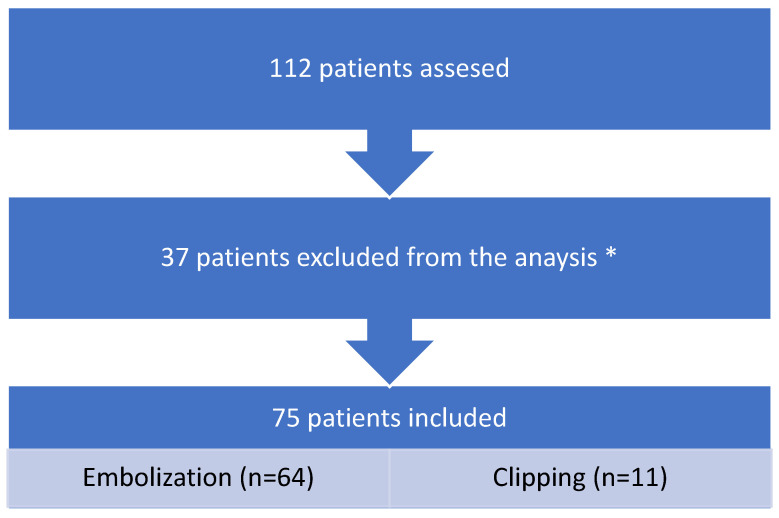
STROBE flow chart. * did not meet the criteria.

From December 2023 to October 2024, 112 patients with unruptured intracranial aneurysms were eligible for treatment. Seventy-five patients (67.0%) who met the inclusion criteria were included in the further analysis. The most common reasons for exclusion were: subarachnoid hemorrhage (*n* = 18), AVMs (*n* = 7), atherosclerosis of cerebral arteries (*n* = 9), and lack of complete clinical data (*n* = 3). The detailed information about inclusion and exlusion cryteria are present in Figure 1 and listed below:

Inclusion criteria:

We investigate all patients diagnosed with unruptured intracranial aneurysms.

Criteria for exclusion from the study group:

- Patients with subarachnoid hemorrhage;
- Arteriovenous malformations;
- Atherosclerosis of cerebral arteries;
- Patients diagnosed with cold aneurysm underwent angiography only.

### 2.2. Tools

The study used a diagnostic survey method. This original questionnaire included socio-demographic data and clinical data obtained electronically from patients’ medical records, in compliance with the principles of personal data protection. The assessment was carried out at established time points, i.e., on the 1st and 3rd day after the procedure. The measurements obtained in the study were classified according to the following scales.

The index of patient categorization can be compared to the level of care assessment (LOC). The categorization indicator is regulated by Polish law and based on care determinants such as mobility, scope of hygiene activities, excretion, nutrition, and orientation regarding one’s health situation, enabling the determination of the level of assistance and intensity of care the patient requires. Category I—minimum care, which mean that patient is usually independent, walk on his own and do not require a special diet; category II—moderate care, patient requires some nursing assistance in some activities, category III refers to the patients who need constant nursing support in all activities, category IV—patient in critical condition, must be continuously monitored [5,6].

Glasgow Coma Scale (GCS) This scale is used to assess the patient’s level of consciousness. It covers three areas: eye-opening, verbal response and motor response. Each category is evaluated based on specific criteria, and the total score ranges from 3 (most severe condition, no response) to 15 (full consciousness and minimal neurological deficits) [6].

*The Modified Rankin Scale (mRS)* evaluates a patient’s degree of disability and level of independence, primarily following a stroke. The tool uses a 7-point scale, ranging from 0 (no symptoms) to 6 (death). Higher scores indicate greater disability, with scores from 3 to 5 representing functional dependence [7].

*The Waterlow Scale* is used to assess the risk of pressure ulcers in patients. It encompasses various risk factors, including mobility, skin condition, nutrition, and comorbidities. The total score obtained allows for the adjustment of appropriate anti-decubitus prophylaxis [8].

### 2.3. Ethical Approval

This study was conducted in accordance with the Declaration of Helsinki and its subsequent revisions. The study protocol was approved by the Independent Bioethics Committee at the Medical University of Gdańsk (approval number KB/30/2025) on 11 February 2025. This retrospective research was based on patients’ medical records. Such research does not involve medical intervention but does entail ethical considerations, including respect for patient rights in accordance with good clinical practice. The study is cognitive in nature. Informed consent for participation was not required due to the retrospective design and in accordance with local regulations. Patients admitted to Polish hospitals are informed that research may be conducted using their anonymized data, with no personal identifiers disclosed. All personal data are anonymized and treated as confidential according to internal hospital policy.

### 2.4. Statistical Analysis

Descriptive statistics methods, namely number (n) and percentage (%), were used to present the results obtained on a nominal and ordinal scale. In the statistical analysis, the χ2 test of independence was used to assess the relationship between the studied variables on a nominal and ordinal scale. To present the results obtained on a quantitative scale, descriptive statistics methods were used, i.e., arithmetic mean (M), median (Me), standard deviation (SD), quartile range (IQR), minimum (Min), and maximum (Max). The Wilcoxon pairwise order test assessed the difference between the two measurements. The U Mann–Whitney test was used to determine the difference between the two groups. The Kruskal–Wallis Rank ANOVA test was used to assess statistically significant differences between multiple groups, and the Dunn test was used as a post hoc test to evaluate which groups had statistically significant differences.

## 3. Results

### 3.1. Characteristics of the Study Group

This study was conducted on 75 patients hospitalized in the Department of Neurosurgery. Analyzing the gender structure of the respondents, the largest group was women, comprising 78.67% (n = 59), with an age range of 66–75 (n = 23; 30.67%). The aneurysms were located in various parts of the body. The largest group of patients was patients with aneurysms located in the internal carotid artery (n = 31; 41.33%), anterior communicating artery (n = 19; 25.33%), and middle artery (n = 19; 25.33%). Taking into account the clinical condition of the patients, embolization was used in 85.33% (n = 64). A detailed summary of the analyzed results is presented in Table 1.

When analyzing risk factors among patients with brain aneurysms, the most frequently indicated factor was female gender (*n* = 52, 69.33%) and hypertension (*n* = 46, 61.33%). Women constituted 78.67% of the study group (*n* = 59). Female gender was also identified in 69.33% of cases as a recognized epidemiological risk factor for aneurysm occurrence.

**Figure 2:**
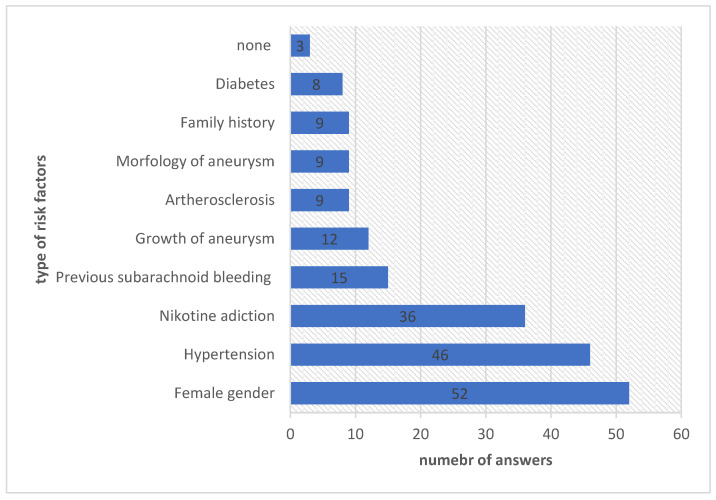
The analysis of risk factors among patients with aneurysms.

Nicotine addiction affected 36 patients (48.00%), and previous subarachnoid bleeding from a ruptured aneurysm—15 of the analyzed cases (20.00%) (Figure 2).

The discrepancy between these values results from different denominators: the first re-flects sex distribution in the cohort, the second—the frequency with which female sex was recorded as a risk factor in clinical documentation.

**Figure 3:**
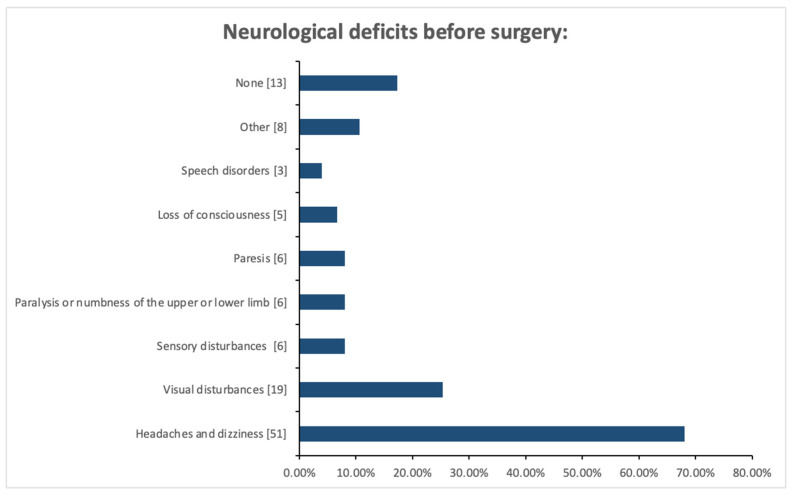
Neurological deficits before medical procedure among patients with cerebral aneurysm.

**Figure 4:**
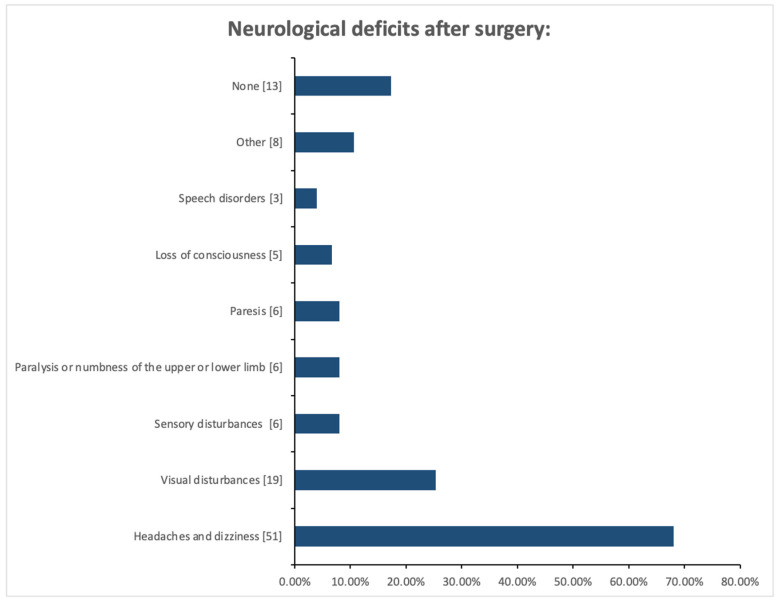
Neurological deficits after medical procedure among patients with cerebral aneurysm.

The results in the figure (Figure 3) illustrate the types of neurological deficits occurring in patients before the procedure. The most frequently reported neurological deficit before the procedure was headache and dizziness (*n* = 51, 68%). Vision disorders occurred in 19 people (25.33%), and paresis, paralysis, and sensory disorders occurred in 6 patients (8.00%). The results in Figure 4 illustrate the neurological deficits reported by patients after the procedure. After the procedure, 40 people (53.33%) had no neurological deficits. Other reported problems were headaches and dizziness (*n* = 19; 25.33%), visual disturbances (*n* = 12; 16%) and sensory disturbances (*n* = 8; 10.67%).

### 3.2. The Influence of the Type of Procedure (Embolization vs. Clipping) on the Functional Assessment of Patients on the 1st and 3rd Day After the Procedure, Measured Using the Barthel and Rankin Scale

**Figure 5:**
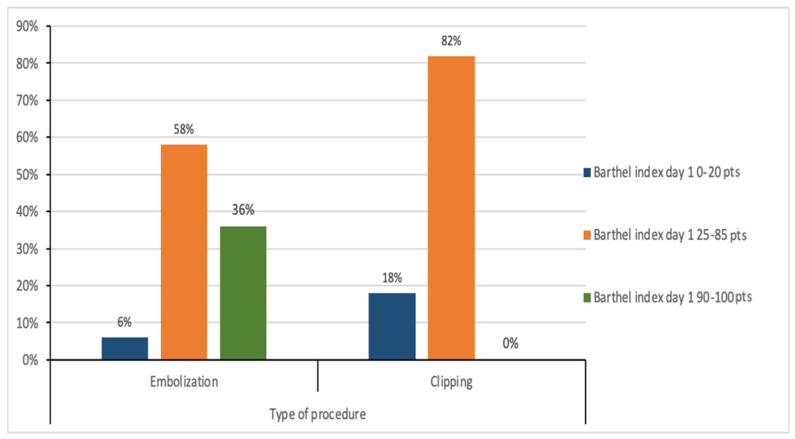
Relationship between the type of surgery and the Barthel index on the first day after surgery.

The results presented in Figure 5 illustrate the relationship between the type of procedure and the Barthel Index on the first day after the procedure. In the group of patients undergoing embolization, four people (6.25%) achieved a Barthel index of 0–20 points, 37 patients (57.81%) achieved an index of 25–85 points, and 23 people (35.94%) achieved an index of 90–100 points. In the group of patients undergoing clipping, two people (18.18%) achieved a Barthel index of 0–20 points, nine patients (81.82%) obtained an index of 25–85 points, and no person (0.00%) achieved an index of 90–100 points. The chi-square test showed a statistically significant relationship between the type of surgery and the Barthel index on the first day after surgery (χ^2^ = 9.42; *p* ≤ 0.01).

**Figure 6:**
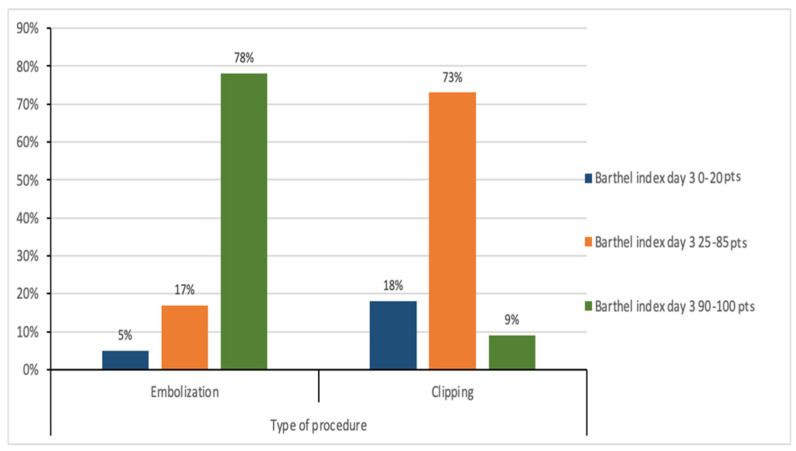
Relationship between the type of surgery and the Barthel index on the third day after surgery.

The results presented in Figure 6 show the relationship between the type of procedure and the Barthel index on the third day after the procedure. In the group of patients undergoing embolisation, 3 people (4.69%) obtained the Barthel index at the level of 0–20 points, 11 people (17.19%) obtained the index in the range of 25–85 points, and 50 people (78.13%) achieved an index of 90–100 points. In the group of patients undergoing clipping, two people (18.18%) achieved a Barthel index of 0–20 points, 8 people (72.73%) achieved an index of 25–85 points, and 1 person (9.09%) achieved an index of 90–100 points. The chi-square test showed a statistically significant relationship between the type of surgery and the Barthel index on the third day after surgery (χ^2^ = 20.09; *p* ≤ 0.001).

**Figure 7:**
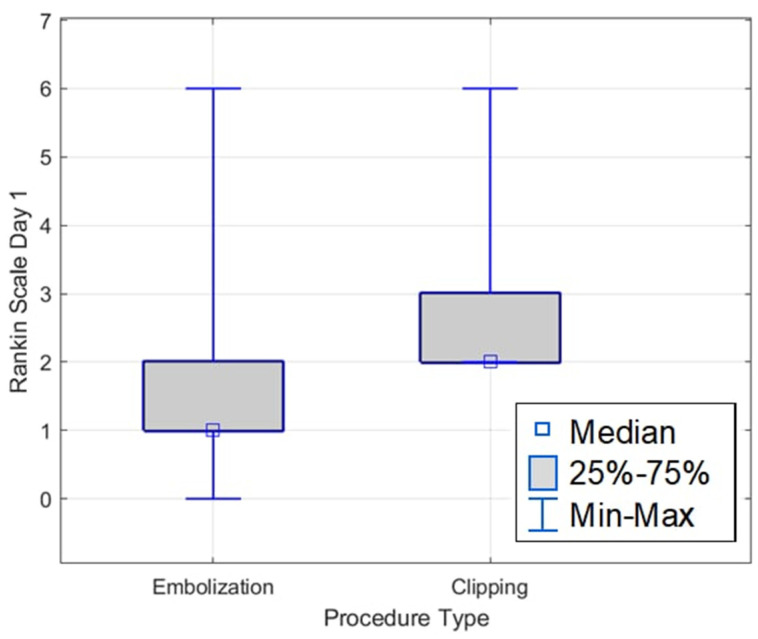
Rankin Scale on the first day, depending on the type of procedure.

The average Rankin Scale value on the first day after embolisation was 1.53 (SD = 1.07), and the median was 1.00 (IQR = 1.00). The lowest value was 0.00, and the highest was 6.00. However, in the group of patients who underwent clipping, the mean value was 2.73 (SD 1.19), and the median was 2.00 (IQR = 1.00). Statistically, significantly lower Rankin Scale values were found on the first day in the group of patients after embolisation than after clipping (Z = −3.49, *p* ≤ 0.001) (Figure 7).

**Figure 8:**
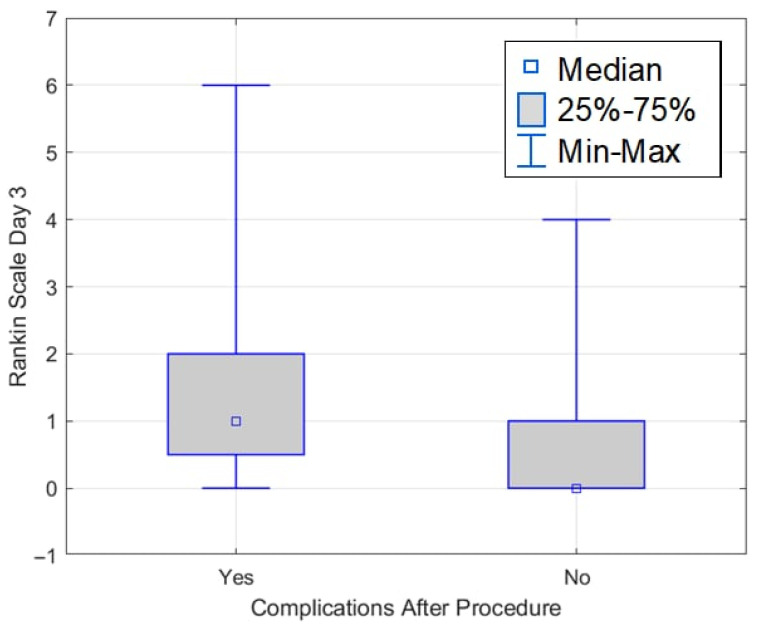
Rankin Scale on the third day, depending on the type of procedure.

The average Rankin Scale value on the third day after embolisation was 0.88 (SD = 1.34), and the median was 0.50 (IQR = 1.00). The lowest value was 0.00, and the highest was 6.00. However, in the group of patients who underwent clipping, the mean value was 2.00 (SD = 1.48), and the median was 2.00 (IQR = 1.00). Statistically, significantly lower Rankin Scale values were found on the third day in the group of patients after embolisation than after clipping (Z = −3.13, *p* ≤ 0.01) (Figure 8).

**Figure 9:**
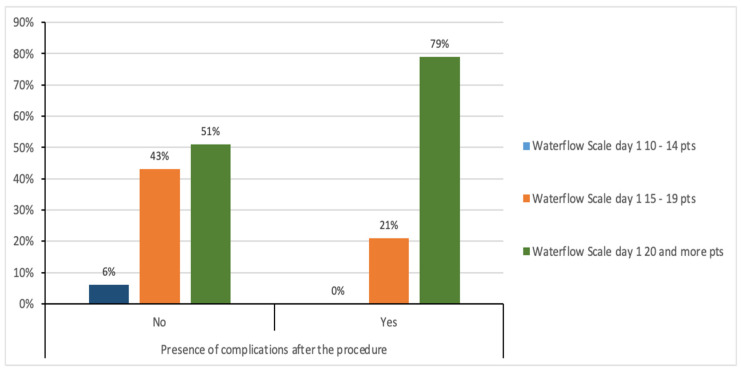
Relationship between the Waterlow scale value on the firsth day and complications after the procedure.

The results presented in Figure 9 show the relationship between the Waterlow scale value on the first day, and the presence of complications after the procedure. In the group of patients with complications, none of the analyzed cases achieved a score of 10–14 points, 6 patients (21.43%) achieved a score of 15–19 points, and 22 people (78.57%) had a score of 20 points or more. In the group without complications, 3 people (6.38%) achieved a score of 10–14 points, 20 people (42.55%) achieved a score of 15–19 points, and 24 people (51.06%) had a score of 20 points and more. The chi-square test showed a statistically significant relationship between the Waterlow scale on the first day and the presence of complications after the procedure (χ^2^ = 7.33; *p* ≤ 0.05). Patients with a higher Waterlow score are more likely to have complications on the first day after the procedure.

**Figure 10:**
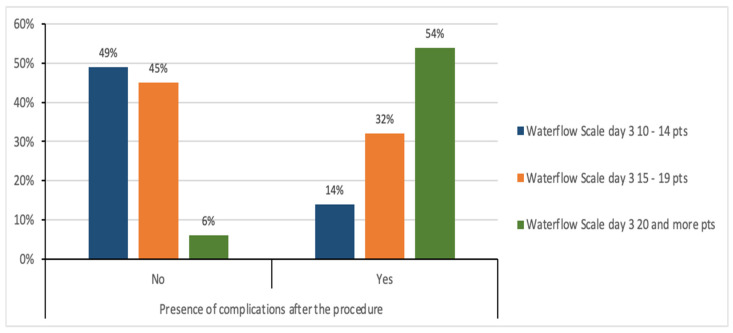
Relationship between the Waterlow scale value on day 3 and complications after the procedure.

Figure 10 shows the relationship between the Waterlow scale value on day 3 and the presence of complications after the procedure. In the group of patients who did not experience any complications after the procedure, 23 people (48.94%) had a score of 10–14 points on the Waterlow scale, 21 people (44.68%) had a score of 15–19 points, and 3 people (6.38%) had a score of 20 points or more. In the group of patients who experienced complications after the procedure, 4 people (14.29%) had a score of 10–14 points, 9 people (32.14%) had a score of 15–19 points, and 15 people (53.57%) had a score of 20 points or more.

The chi-square test showed a statistically significant relationship between the Waterlow scale score on day 3 and the presence of complications after the procedure (χ2 = 23.58; p ≤ 0.001). Patients with a higher Waterlow score are more likely to have complications on the third day after the procedure.

### 3.3. Dynamics of Postoperative Pain (Nrs) During Follow-Up

**Figure 11:**
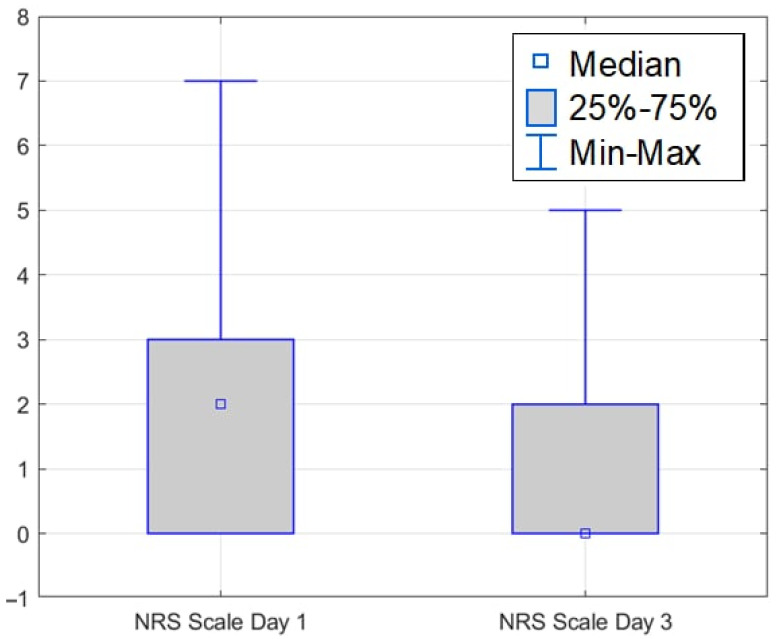
NRS scale on the 1st and 3rd days after surgery.

The average NRS score on the first day was 2.00 (±1.97), while on the third day the average score was 0.89 (±1.32). It was shown that the mean NRS score decreased by 1.11 (±1.64). The decrease was found to be statistically significant (Z = 4.87; *p* ≤ 0.001) (Figure 11).

## 4. Discussion

The effectiveness of treatment is assessed not only by the immediate success of the procedure but also by the patient’s subsequent functioning. The early postoperative period is crucial for evaluating functional recovery and identifying potential complications. The study showed that intracranial aneurysms most often occur in women aged 66–75. The presented research results are consistent with epidemiological analyses [9]. The choice of treatment was not randomized and depended on aneurysm morphology, patient condition, and local treatment preferences. Therefore, causal interpretation of the observed differences should be made with caution. Many factors influence treatment decisions, including the patient’s overall condition, age, location and morphology of the aneurysm. Endovascular embolization has become the preferred method of therapy over the years [3,9,10]. Embolization was the most frequently used procedure in patients of the study group (85.33%). Endovascular embolization is often preferred in elderly patients or aneurysms with favourable morphology, whereas surgical clipping may be more common in MCA bifurcation aneurysms or those with wide necks. These factors can affect recovery trajectories and may partly explain the observed differences in early functional outcomes [11,12]. The study showed a statistically significant difference in the functional assessment of patients (measured by the Barthel and Rankin scales) depending on the method used. The analysis confirmed that the embolization procedure leads to a faster and better recovery compared to the clipping procedure.

The detection of an aneurysm in patients was most often associated with symptoms that made everyday functioning difficult, such as headaches and dizziness. Oki-Kwan, in his work, states that headache is the main symptom reported by patients. However, the cause-and-effect mechanism is not fully known. Pain from an unruptured cerebral aneurysm may occur if it develops in the proximal cerebral arteries, where pain fibers are present. An individualized approach to pain treatment, following the analgesic ladder, is important to enable patients to function in everyday life as much as possible [13,14].

In the study, pain was assessed using the NRS. The mean NRS score was 2.00 (±1.97) on the first postoperative day, indicating moderate pain. By day 3, the mean pain score decreased significantly to 0.89 (±1.32). This reduction of 1.11 (±1.64) points was statistically significant (Z = 4.87, *p* ≤ 0.001), demonstrating effective pain management. Pain management in patients after neurosurgery is a critical issue that requires a multidisciplinary approach and careful consideration of many factors [13].

In the study by Obuchowska et al., ocular disorders were among the most important symptoms indicating an aneurysm. In 5 people from the group of 16 patients with unruptured aneurysms, splitting occurred immediately after the procedure [15]. In our study, double vision occurred among 12 patients before and after the procedure. There was a statistically significant relationship between a higher Waterlow score on the first postoperative day and the occurrence of complications. Patients with higher scores were more likely to develop complications. Notably, in the complication group, no patient scored 10–14; 21.43% scored 15–19, and 78.57% scored 20 or higher. In the group without complications, 6.38% of patients scored 10–14 points, 42.55% 15–19 points, and 51.06% 20 or more points. The significant association continued on day 3, with higher Waterlow scores associated with a greater likelihood of complications. In the group without complications, 48.94% scored 10–14 points, 44.68% scored 15–19 points, and 6.38% scored 20 or more. In the group with complications, 14.29% obtained 10–14 points, 32.14% 15–19 points, and 53.57% 20 or more points. The Waterlow score was a valuable predictor of postoperative complications in these patients. A higher Waterlow score on both day 1 and day 3 indicated a significantly increased likelihood of complications. This suggests that the Waterlow Score may be valuable in identifying patients who require more intensive monitoring and preventive measures [16,17,18]. The functional assessment was limited to early postoperative days (1 and 3). These time points are clinically crucial for nursing care and monitoring, but may not reflect functional independence at discharge or 30–90 days postoperatively.

The presented research results are among the first conducted in Poland using the tools mentioned above.

## 5. Strengths and Limitation

This study also has some limitations. The first one is that the study had only single-one retrospective character, so we cannot generalize the results to bigger populations. We delved more deeply in clinical characteristic of the patient (morphological assessment like size and shape of the structure) because our aim was to present the nursing issues in the early functional assessment. That is why in the result section we focused not only on the Rankin Scale but also the Waterlow and pain tool. The strengths of our study rely on standardized postoperative assessments using validated scales and a clearly defined perioperative period.

We strongly believe that apart of limitations, our results can be useful in everyday practice, especially in planning the proper number of nurses per shift. Our results proved that after surgery patient experience many worrying symptoms like hematomas, bleeding at the side of surgery, headaches and pain. Those should be noticed and cured as fast as possible on the basis not only therapeutic intervention but also well-thought-out nursing care plan. There is a high need to lead more nursing research in that area, not only in the first few hours after the surgical treatment but also as a follow up process to increase patient life quality.

## 6. Conclusions

Based on a review of the literature and our own research findings, the following conclusions can be drawn:

In most analyzed cases, there were no significant changes in patients’ functional status between days 1 and 3, as measured by the Barthel and Glasgow scales. The results demonstrated a relationship between higher Waterlow scores on the first and third days after the procedure and the occurrence of complications; however, these findings require further investigation in larger patient cohorts with extended follow-up.

Detailed analysis revealed that the largest patient group consisted of women aged 66–75. The internal carotid artery was the most common site of an aneurysm. Patients treated with embolization showed better functional outcomes on the Barthel and Rankin scales on days 1 and 3 post-procedure than those who underwent clipping. No statistically significant association was found between aneurysm location and the incidence of neurological deficits following the procedure.

The most frequent risk factors identified in patients with brain aneurysms were female sex, hypertension, and nicotine dependence. The most common complications observed after surgery were hematomas or bleeding at the injection site and headaches. Patients experience pain after surgery, but this symptom (evaluated in NRS scale) decreased significantly between the 1st and 3rd day after the procedure.

## 7. Implications on Nursing Practice

Patients experience pain after surgery, but this symptom (evaluated in NRS scale) decreased significantly between the 1st and 3rd day after the procedure.

This study evaluates the functional outcomes of patients with intracranial aneurysms treated surgically by either clipping or embolization. The analyses confirmed that higher Waterlow pressure ulcer risk assessment scores on the first and third days after surgery were associated with an increased risk of postoperative complications. The Waterlow scale may therefore serve as a valuable indicator in planning nursing care for patients following either surgical method.

The study also identifies the most common risk factors among individuals diagnosed with intracranial aneurysms. In the future, it would be beneficial to develop comprehensive health and prevention programs focused on the early detection of intracranial aneurysms and on reducing modifiable risk factors, such as nicotine dependence and hypertension, particularly in women.

## Figures and Tables

**Table 1:** Detailed socio-demographic characteristics of the studied group.

*Variable*	Total (n = 75)	Embolization (n = 64)	Clipping (n = 11)	*p*-Value
***Age, mean ± SD (years)***	57.2 ± 9.3	57.8 ± 8.6	54.3 ± 10.8	0.28
***Female sex, n (%)***	59 (78.7%)	50 (78.1%)	9 (81.8%)	0.78
***Hypertension, n (%)***	46 (61.3%)	38 (59.4%)	8 (72.7%)	0.41
***Diabetes mellitus, n (%)***	11 (14.7%)	9 (14.1%)	2 (18.2%)	0.71
***Active smoking, n (%)***	25 (33.3%)	20 (31.3%)	5 (45.5%)	0.36
***Previous SAH, n (%)***	9 (12.0%)	6 (9.4%)	3 (27.3%)	0.12
***Baseline neurological deficit, n (%)***	7 (9.3%)	5 (7.8%)	2 (18.2%)	0.27
***Aneurysm size, mean ± SD (mm)***	5.8 ± 2.4	5.6 ± 2.3	6.4 ± 2.7	0.33
***Neck morphology***				
*– Narrow neck (<4 mm)*	49 (65.3%)	45 (70.3%)	4 (36.4%)	0.04 *
*– Wide neck (≥4 mm)*	26 (34.7%)	19 (29.7%)	7 (63.6%)	
***Aneurysm location***				
*– ICA (internal carotid artery)*	23 (30.7%)	22 (34.4%)	1 (9.1%)	0.08
*– MCA (middle cerebral artery)*	14 (18.7%)	8 (12.5%)	6 (54.5%)	0.01 *
*– ACoA (anterior communicating artery)*	21 (28.0%)	18 (28.1%)	3 (27.3%)	0.95
*– **PCoA (posterior communicating artery)***	11 (14.7%)	10 (15.6%)	1 (9.1%)	0.60
*– Other*	6 (8.0%)	6 (9.4%)	0 (0%)	0.34

*—statistically significant.

## Data Availability

The data can be provided from the corresponding author upon reasonable request.
